# Application of Hydrophilic Interaction Liquid Chromatography for the Quantification of Flavonoids in *Genista tinctoria* Extract

**DOI:** 10.1155/2016/3789348

**Published:** 2016-06-28

**Authors:** Aleksandra Sentkowska, Magdalena Biesaga, Krystyna Pyrzynska

**Affiliations:** Department of Chemistry, University of Warsaw, Pasteura 1, 02-093 Warsaw, Poland

## Abstract

Hydrophilic interaction chromatography (HILIC) was employed to investigate chromatographic behavior of selected flavonoids from their different subgroups differing in polarity. Chromatographic measurements were performed on two different HILIC columns: unmodified silica (Atlantis-HILIC) and zwitterionic sulfoalkylbetaine (SeQuant ZIC-HILIC). Separation parameters such as content and type of organic modifier were studied. On ZIC column retention factors were observed to be inversely proportional to the buffer content in the mobile phase, which is the typical partitioning mechanism. In the case of bare silica column more or less apparent dual retention mechanism was observed, depending on the water component content in the mobile phase. ZIC-HILIC showed better selectivity (in comparison to silica column) with the detection limit of 0.01 mg/L (only for rutin was 0.05 mg/L). Finally, this chromatographic procedure was validated and applied for the determination of some flavonoids in *Genista tinctoria* L. extract.

## 1. Introduction

Hydrophilic interaction chromatography (HILIC) was first introduced by Alpert in 1990 [1] and since then has attracted increasing attention. HILIC seems to be a missing tool in the analysis of polar and ionized compounds, which are too strongly retained on polar stationary phases used in normal phase mode and/or are poorly separated when reserved phase mode is applied [2–5]. The retention mechanism in HILIC seems to be complex. It was first suggested that the partition of analyte between the organic-rich mobile phase and the immobilized water-rich layer at the surface of the stationary phase is the main retention mechanism. Recently, various studies have indicated that HILIC involves a complex multiparametric retention process composed of partitioning or adsorption via hydrogen bonding or other dipole-dipole interactions as well as electrostatic interactions with bonded ionic groups [4–7]. Different interactions that contribute to the overall HILIC retention are dependent on the column type and the polarity and ionization of the analytes as well as the mobile phase composition.

Many stationary phases have been applied in HILIC mode [8, 9]. Most of them are silica based materials, which can be uncoated and derivatized with different kinds of functional groups. Acetonitrile (ACN), as a polar aprotic solvent, is proven to be the best organic solvent for HILIC. Alcohols such as methanol, ethanol, or isopropanol are less used; however, they can compete for active polar sites on a stationary surface and analytes, whose retention is based on strong hydrogen bonding [10–12].

*Genista tinctoria *L. (also named dyer's greenweed, waxen wood, and “Royal Gold”) is a flowering plant species of the Fabaceae family. It is a small shrubby plant with narrow, pointed leaves and yellow flowers, usually growing in meadows, pastures, and fields.* Genista tinctoria* is famous due to its application as a yellow dye obtained from the whole plant, but especially from the flowers and young shoots. Its decoction is used in kidney disease, urolithiasis, and inflammation of the kidneys [13]. It can also be used as a diaphoretic, as well as for the treatment of gout, rheumatism, and ascites water. Previous studies showed that the extract of this plant exhibited high antioxidant activity due to the high presence of polyphenolic compounds [14]. Flavonoids comprise the most abundant class of plant polyphenols. In recent years they have been widely studied due to their wide spectrum of action in human body [15]. Recent epidemiological studies show that a diet rich in flavonoids reduces the risk of incidence of cardiovascular disease and cancer [16, 17]. Reversed phase liquid chromatography (RP-HPLC) is a commonly applied separation technique for quantification of flavonoids in different kinds of samples [18–20].

The objective of this work was to investigate chromatographic behavior of several flavonoids onto two HILIC stationary phases—unmodified silica and zwitterionic sulfobetaine. The compounds used for the experiments differ in polarity. Acetonitrile and methanol were applied as the main components of mobile phases. In acetonitrile or methanol-rich mobile phase, spraying conditions improve and enhance the efficiency of desolvation and ionization in the electrospray ion source, which can provide increasing sensitivity with respect to reverse phase conditions. Finally, the potential of hydrophilic interaction liquid chromatography was used in the analysis of* Genista tinctoria* extract.

## 2. Materials and Methods

### 2.1. Reagents

Water was obtained from a Milli-Q water purification system (Millipore, Bedford, MA, USA). Acetonitrile (ACN) and methanol (MeOH) were of HPLC grade from Merck (Darmstadt, Germany).

The commercial standards of flavonoids were purchased from Sigma (Steinheim, Germany). The stock solutions of these individual compounds were prepared at 100 *μ*g mL^−1^ concentration level in acetonitrile. The 1 *μ*g mL^−1^ of standard solutions used in the study was prepared by dilution by appropriate solvent composition. All solutions were filtered through PTFE 0.45 *μ*m membrane filters (Millipore) and degassed prior to use.

### 2.2. Instrumentation

Chromatographic analysis was performed with the Shimadzu LC system consisting of binary pumps LC20-AD, degasser DGU-20A5, column oven CTO-20AC, autosampler SIL-20AC, and 3200 QTRAP Mass spectrometer (Applied Biosystem/MDS SCIEX). The MS system was equipped with the electrospray ionization source (ESI) operated in negative-ion or in positive mode. ESI conditions were as the following: the capillary temperature 450°C, the curtain gas at 0.3 MPa, the auxiliary gas at 0.3 MPa, and the ionisation mode source voltage of 4.5 kV. Nitrogen was applied as the curtain and auxiliary gas. For each compound the optimum conditions of Multiple Reaction Mode (MRM) were determined in the infusion mode. Standard solutions were infused into the electrospray source via 50 *μ*m i.d. PEEK capillary employing the Harward Apparatus pump at 10 *μ*L/min. Continuous mass spectra were obtained by scanning* m/z* from 50 to 650.

Compounds were separated on two chromatographic columns: Atlantis-HILIC column (100 × 2.1 mm, 3.0 *μ*m) from Waters (Milford, MA, USA), and SeQuant*™* ZIC-HILIC column (100 × 2.1 mm, 3.5 *µ*m) from Merck (Darmstadt, Germany). The mobile phase containing ACN/ammonium formate or MeOH/ammonium formate mixture was delivered at 0.2 mL/min in isocratic or gradient mode. The analytes were identified by comparing retention time and* m/z* values obtained by MS and MS^2^ with the mass spectra [21].

### 2.3. Sample Preparation

Dried plant of* Genista tinctoria *L. was purchased from Botanical Garden in Powsin, Poland. Herbs were collected in the 2015 season and air dried at room temperature. 0.2 g of air-dried plant was extracted with 30 mL of water/methanol (40 : 60, v/v) solvent and heated in a water bath for 30 min [22]. After cooling to room temperature, three of 1 mL samples of plant extract were dried under inert gas atmosphere. Then 1 mL of acetonitrile was added, filtered, and injected to HPLC system. The results are expressed as mean ± standard deviation based on at least three independent determinations.

### 2.4. Partition Coefficient Determination

The log⁡*P* values for flavonoids were calculated using Molinspiration program (available* online* at http://www.molinspiration.com/cgi-bin/properties) from Molinspiration Cheminformatics (Slovensky Grob, Slovakia). This program employs fragmental analysis of chemical structure for prediction the value of log⁡*P*. The obtained results are presented in Table 1.

## 3. Results and Discussion

**Figure 1:**
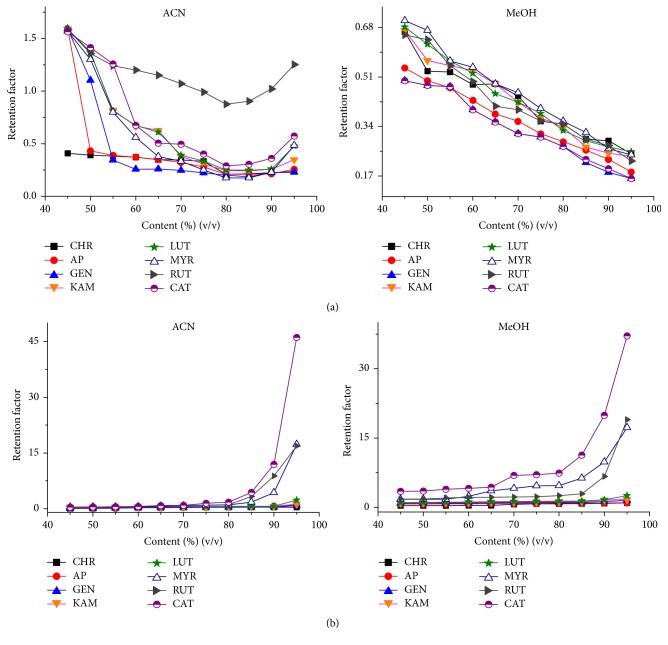
The retention factor as a function of ACN or MeOH content in the mobile phase. (a) Bare silica column; (b) ZIC column.

The effect of organic solvent content in the mobile phase on the retention of flavonoids was investigated using acetonitrile and methanol. The study was carried out in the presence of ammonium formate at pH 7.0, which refers to the aqueous portion of the mobile phase. It should be noticed that the apparent pH value of the mobile phase containing high percentage of organic solvent is different from that of the water component [23, 24]. At the acidic pH, the acidity of such mobile phase is likely higher than the aqueous acid or buffer solution applied for eluent preparation. Mobile phase pH plays a key role in retention due to its impact on ionization state of both analytes and stationary phase. Silanol groups are weak acids and become deprotonated (negatively charged) at higher pH values. Positively charged compounds can have electrostatic interaction with such groups and can result in stronger retention. Therefore, it is important to consider the impact of mobile phase pH on the stationary phase and the ionization state of the analytes. The p*K*_*a*_ values of our analytes are in range of 6.4–7.1. Thus at pH 7.0 they exist in the equilibrium between the charged and the neutral forms. The obtained retention factors were plotted against the organic solvent content in the range of 40–95% (v/v) as shown in Figure 1.

**Figure 2:**
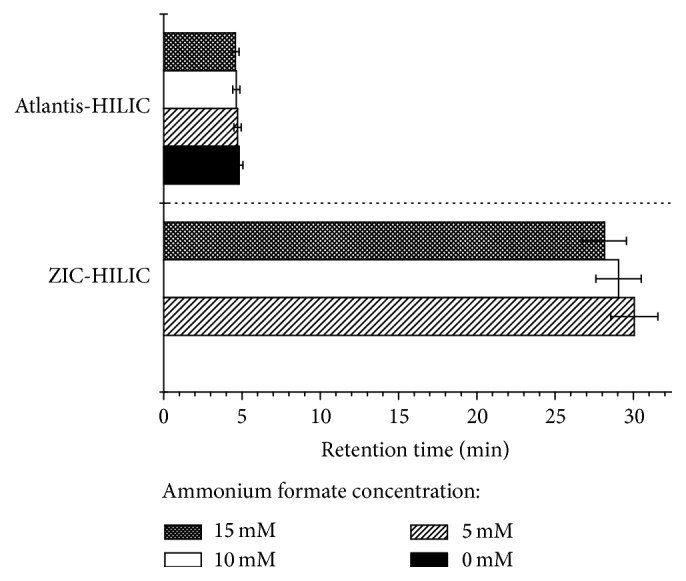
The retention time of rutin at different concentration of ammonium formate (95% ACN v/v pH 7).

The higher content of both applied organic solvent in the mobile phase enhances the retention of studied flavonoids on ZIC-HILIC column. At increasing concentration of acetonitrile, water is adsorbed more strongly on the surface of the polar stationary phase. The more hydrophilic the analytes are, the more the partitioning equilibrium is shifted towards the adsorbed water layer on the stationary phase, and the more analytes are retained. It can be clearly seen on the example of rutin. For this flavonoid the retention factor increases rapidly with the increase of ACN. Its retention time is longer than 120 min, when mobile phase containing 95% of ACN is applied. Such a high value of retention factor for rutin can be shortened when ACN is replaced with MeOH (Figure 1). The layer formed onto stationary phase is less polar, when methanol is used, which is the main reason for decreasing retention of more polar compounds (myricetin, rutin, and catechin). On the other hand, methanol as a main component of the mobile phase enhances the retention of less polar flavonoids. The use of methanol as a mobile phase component reduces the retention time of the rutin but worsens the separation of other studied flavonoids. In such a situation the application of acetonitrile is necessary. However, the use of ammonium formate (pH 7.0) instead of the water (pH about 7.0) as an inorganic component of mobile phase enables shortening rutin retention time without losing the separation of other compounds (Figure 2). The biggest differences in retention times of rutin are shown on ZIC-HILIC column when 5 mM ammonium formate is used instead of water in the eluent. Shortening of the retention time from longer than 120 min (not indicated on the graph) to about 30 min is obtained. A further increase in buffer concentration also reduces the retention time of rutin but the differences are not so considerable. A similar correlation was observed for silica column, but due to the low retention of rutin on this stationary phase, the differences in retention times are insignificant. The presence of salt in the eluent shields electrostatic interactions, both attractive and repulsive. As it was reported before [25], the presence of salt in the mobile phase slightly increases the retention for hydrophobic aglycones. On the other hand, the retention factors of more polar compounds such as rutin decrease when higher concentration of buffer is applied. This fact suggests that repulsive interactions between more polar flavonoid and stationary phase surface play a key role in the retention mechanism.

Generally, the bare silica packing consistently delivered lower retention factors. The U-shaped curves are obtained when solute retention factors are plotted versus the ACN content (Figure 1). This stationary phase showed more or less apparent dual retention mechanism, HILIC at the ACN content higher than 80%, and RP at lower content. This tendency was also observed for toluene, which was applied as the void time marker. In the contrary, the retention of all studied flavonoids decreased with increasing content of methanol in the mobile phase. Methanol, with its strong ability for hydrogen bonding, can more effectively compete for the active sites on the stationary phase, perturbing the formation of water layer and replacing water molecules due to similar polarity, and thus leading to the reduced retention [26]. Buszewski et al. [27] reported that methanol may be adsorbed near the silica surface through hydrogen bonding with residual accessible silanols, while acetonitrile can react with silanol groups by dipole-dipole interactions and the amount of adsorbed ACN molecules is about four times higher than for MeOH.

**Figure 3:**
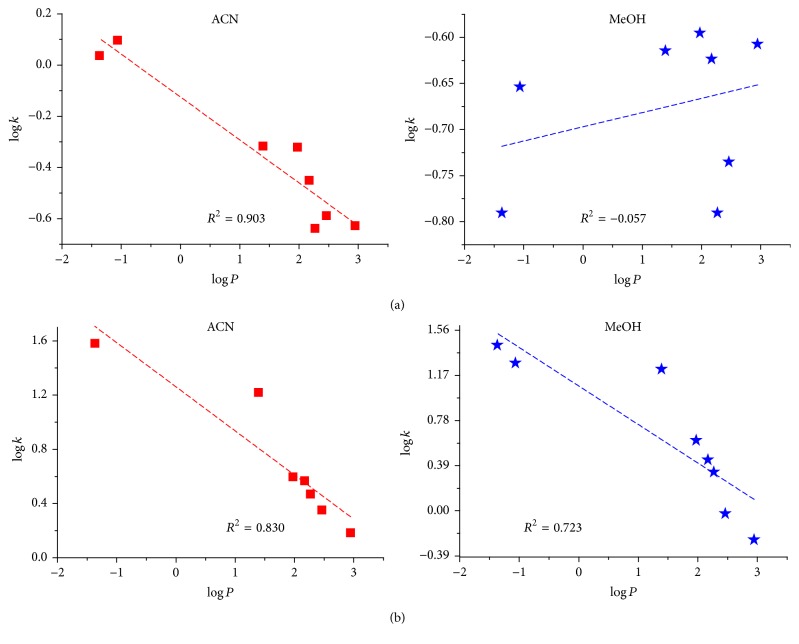
The relationship between log⁡*k* of flavonoids and log⁡*P*. Mobile phase: 95% ACN/ammonium formate or 95% MeOH/ammonium formate. (a) Bare silica column; (b) ZIC column.

Since hydrophilic interactions are one of the main mechanisms which governs the retention in the HILIC mode, the hydrophilic properties of a compound should determine its behavior. We examined the relationships between log⁡*k* and calculated partition coefficient *P* (Table 1), a parameter that relates single solute partitions between polar and nonpolar phase. Formally, the application of log⁡*D*, as opposed to the traditional approach of log⁡*P*, should be taken into consideration for ionized species. However, its value depends on compound p*K*_*a*_ as well as pH under investigation. It is worth noticing that the p*K*_*a*_ for some of the studied flavonols are known to be difficult to predict and their reported values differ from each other about 1-2 p*K*_*a*_ units [28–30]. The retention data were acquired with a mobile phase composed of 95% acetonitrile or methanol and the relations are presented in Figure 3. log⁡*k* is inversely correlated to log⁡*P*, indicating that the stronger retained analytes are those with lower log⁡*P* values, namely, the more hydrophilic analytes.

The elution order of tested flavonoids agrees with their polarity when ZIC column was used. Replacing ACN with MeOH does not result in a change of this retention order. These observations may suggest that retention of analytes is highly dependent on their polarity. According to HILIC theory, the partition of analytes between water enriched layer and less polar eluent is the main retention mechanism. However other interactions such as hydrogen bonding, dipole-dipole, or electrostatic interactions can affect the separation. For ZIC column the correlation coefficients of the relationship log⁡*k* versus log⁡*P* were relatively high for both used organic solvents (0.830 for ACN and 0.723 for MeOH). The correlation coefficients are far from unity, indicating additional influence on the separation mechanism other than partitioning. Thus, it is reasonable to propose a mixed-mode retention mechanism, as recently was proved for zwitterionic stationary phases [31].

In the case of silica column, the elution order obtained for both organic solvents differs from the theoretical one. The biggest changes were observed when MeOH was applied. The retention order is as follows: KAT ~ GEN < AP < RUT < KAM < MYR < CHR < LUT. The most polar compound catechin (based on the values of log⁡*P*) is first eluted while the less polar chrysin is strongly retained on the stationary phase. This may suggest that the retention mechanism under these conditions is much more complicated and it is not only dependent on the hydrophilic partition of the analytes [32].

**Figure 4:**
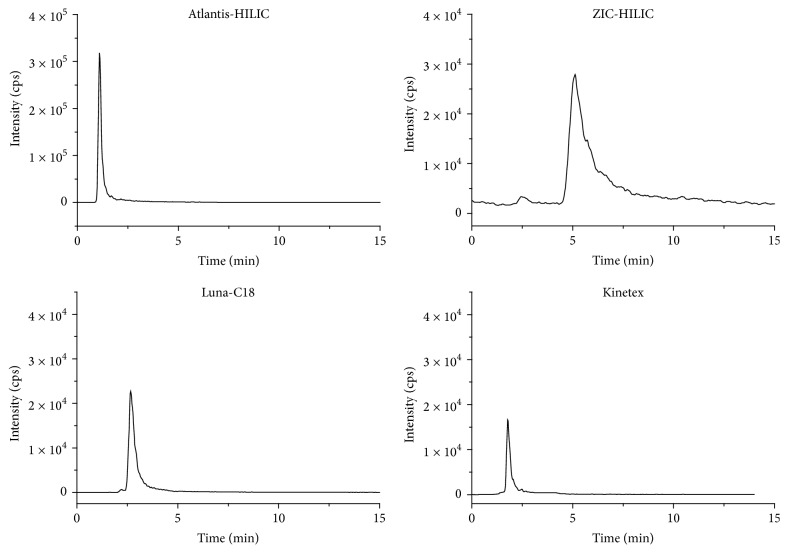
Extracted ion chromatograms of luteolin on HILIC and RP columns under isocratic elution. Eluent for HILIC column-ACN/H_2_O (95/5%, v/v), for Luna-C18 ACN/water (45/55%, v/v), and for C18: ACN/water (20/80%, v/v).

The comparison of HILIC and RP mode was also carried out as reversed-phase mode of the most popular chromatographic mode for flavonoids analysis. Two RP columns were employed—typical fully porous Luna C18 (10 cm × 4.6 mm × 5 Am) and core shell Kinetex C18 (100 × 2.1 mm × 2.6 Am). The composition of the mobile phase in RP mode was ACN/H_2_O (20/80, v/v) to obtain similar retention factors.  Figure 4 presents the MS extracted ion chromatograms of luteolin (SRM 285/133) as a model compound obtained on different HILIC and for comparison RP columns under isocratic elution. The significantly higher sensitivity was observed under HILIC conditions due to much higher content of ACN in the mobile phase (95%, v/v). There were no significant differences in the retention times of luteolin analyzed under HILIC and RP mode. Thus, acetonitrile-rich mobile phase (used in HILIC mode) not only affects the retention factors but also has an impact on the MS detection.

The ZIC-HILIC column showed higher retention of the studied analytes; thus, it was applied for quantification of flavonoids. The binary mobile phase composed of acetonitrile and ammonium formate in the gradient mode starting from 98% (v/v) of ACN has been applied to elute all unknown compounds present in natural samples.

The validation method was performed evaluating linearity of the working range, the limit of detection. The linearity of the working range was evaluated by the construction of calibration curves using linear least squares regression. The calibration curve of six points in triplicate was established in the concentration range of 0.5–25 mg L^−1^. The correlation coefficients (*R* ^2^) were higher than 0.98 for all studied flavonoids (Table 2). The limit of detection was determined by successive dilution of standard solutions until no signal was reliably detected. The value of 0.01 mg L^−1^ with the exception of rutin (0.05 mg L^−1^) was obtained. Accordingly, the limit of quantification (0.03 mg L^−1^) was considered as the lowest concentration of analytes in the standard that can be reproducibly measured with acceptable accuracy and precision. The repeatability of reference standard solution injection (retention times and peak areas) gave the values of the relative standard deviations as 2.9% and 3.1, respectively.

**Figure 5:**
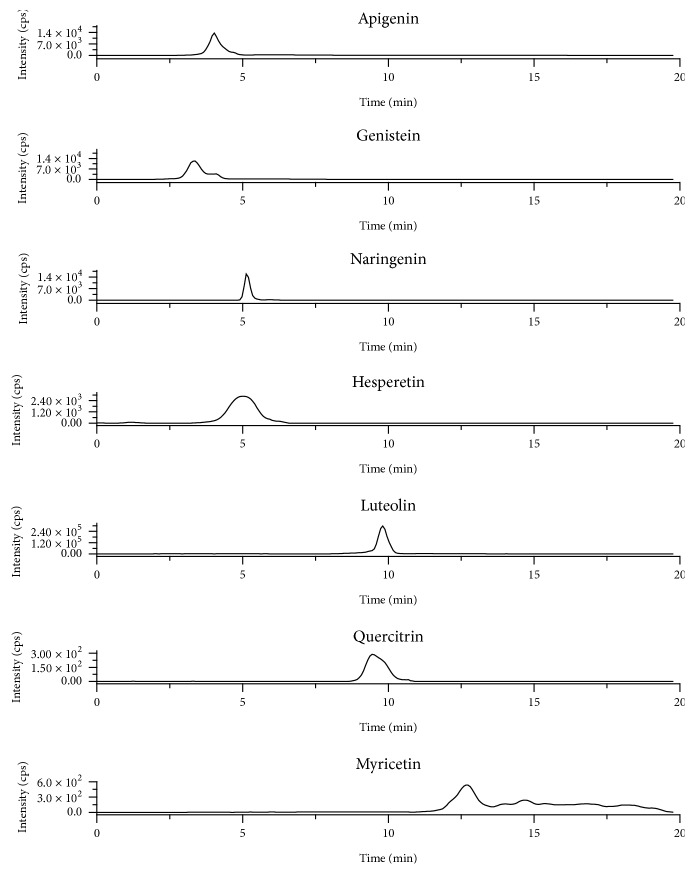
ESI-MS spectra of selected compounds in the* Genista tinctoria* extract. Column: ZIC-HILIC (100 × 2.1 mm, 3.5 *µ*m). Elution gradient: 0–4 min 98% ACN, 6-7 min 90%, 8–8.4 min 80%, 8.4–12 min 50%, and 13–20 min 98%.

Figure 5 shows the obtained ESI-MS/MS chromatograms in SRM mode for selected compounds in the* Genista tinctoria* extract. The identification of the analytes was performed by checking their MS/MS spectra [22]. Obtained results showed high amount of luteolin, genistein, and naringenin (Table 3).* Genista tinctoria* owes its antioxidant properties due to the presence of polyphenolic compounds, mainly flavonoids, such as luteolin, genistein, and apigenin, whose presence was previously reported [33, 34]. The proposed method enabled detection of other flavonoids, such as myricetin, naringenin, apigenin, and quercetrin, which were not reported before in the extract of this plant.

**Figure 6:**
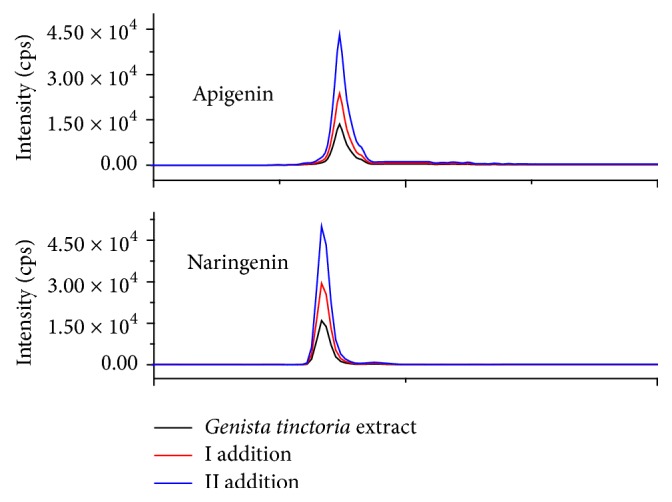
The recovery of apigenin and naringenin in* Genista tinctoria* extract. I: the addition of 0,5 mg of apigenin or 10 mg of naringenin; II: the addition of 1 mg of apigenin or 20 mg of naringenin (mg/g of dried plant in the extract).

To check the accuracy of the method, recovery experiments were performed. Suitable amounts of each detected compound were added to the sample and then analyzed, using proposed method. The obtained recoveries were in ranged from 81 to 95%. The example chromatograms of apigenin and naringenin are presented in Figure 6.

HILIC chromatography can be an alternative separation technique in the analysis of flavonoids. Its potential can be shown mainly when mass spectrometry is applied for the detection. In such case main attention is paid to the limit of detection but not to good separation of the compounds. HILIC mode offers lower limit of detection in comparison to the reversed mode. It is mainly due to the high content of organic solvent in the mobile phase, which enhances the ionisation in the ion source of mass spectrometer. The potential of this technique can be applied at the sample preparation step, mainly when SPE is considered to be used. Analytes can be eluted from SPE column using organic solvent and then directly injected to HPLC system. All these advantages compensate the asymmetry of the peaks and weaknesses of flavonoids separation.

## 4. Conclusions

In this study the effect of organic solvent type in the mobile phase was tested. ACN and MeOH were applied for the separation of flavonoids performed on Atlantis-HILIC and ZIC-HILIC columns. Using MeOH as a mobile phase component results in decreasing of the retention time for more polar compounds. On the other hand it enhances the retention of less polar analytes. The retention factors for flavonoids increase rapidly with the increase of acetonitrile content. The high organic content of the mobile phase enhances the ionisation efficiency in the ion source and simultaneously enhances the sensitivity of detector.

Compared to other chromatographic modes, HILIC could offer also several significant benefits. The highly organic content provides also lower back-pressure to enable fast separations under higher flow rates or to use columns with small particles. HILIC mode is preferred for RP solid phase extracts obtained in a sample preparation step as no evaporation or reconstruction steps are needed. However, HILIC is more influenced by the composition of sample diluent and the retention characteristics are less predictable due to the more complex retention mechanism.

## Figures and Tables

**Table 1:** Polarity of studied flavonoids.

Compound	Abbreviation	log⁡*P*
Chrysin	CHR	2.943
Apigenin	AP	2.463
Luteolin	LUT	1.974
Genistein	GEN	2.268
Kaempferol	KAM	2.172
Myricetin	MYR	1.392
Rutin	RUT	−1.063
Catechin	CAT	−1.370

**Table 2:** Validation parameters for flavonoids analysis. Conditions with ACN/ammonium formate as an eluent.

	LOD [mg·L^−1^]	LOQ [mg·L^−1^]	Slope	*R* ^2^
Apigenin	0,01	0,03	109168	0,983
Genistein	0,01	0,03	2730000	0,994
Hesperetin	0,01	0,03	60385	0,985
Quercetrin	0,01	0,03	448000	0,990
Luteolin	0,01	0,03	1530000	0,993
Myricetin	0,01	0,03	128100	0,990
Naringenin	0,01	0,03	7314000	0,998
Rutin	0,05	0,06	48669	0,980

**Table 3:** The content of flavonoids in *Genista tinctoria* extract.

Compound	Content^*∗*^
Genistein	58.7 ± 1.22
Luteolin	67.5 ± 1.42
Naringenin	53.0 ± 2.60
Quercetin	34.6 ± 1.58
Myricetin	3.86 ± 0.17
Apigenin	3.00 ± 0.15
Quercitrin	1.25 ± 0.06

^*∗*^Values (mg/g of dried plant) are expressed as mean ± SD (*n* = 3).
